# Immune modulatory stem cells represent a significant component of the immune system

**DOI:** 10.3389/fimmu.2025.1543495

**Published:** 2025-03-03

**Authors:** Dmitriy Vladimirovich Karpenko

**Affiliations:** Laboratory of Epigenetic Regulation of Hematopoiesis, National Medical Research Center for Hematology, Moscow, Russia

**Keywords:** stem cells, immune modulatory stem cells, stem system, immune system, evolution, infections

## Introduction

Stem cells are an important compartment supporting tissues with differentiating cells and responding to regeneration demand (1–4). There are interesting evidences that subpopulations of stem cells migrate to developing organs and tissues during embryogenesis, but do not directly contribute to development (5–10). They persist to provide support as stem cells of the adult organism. If more cells would have proliferative and differentiation potential of stem cells, it would be good for regeneration. However, it should be balanced with risks of mutations and oncogenesis. A self-maintaining, highly proliferative cell would need fewer changes to become a cancer cell. The quiescence of stem cells with high proliferative potential could be a solution to place them evolutionarily further away from cancer cells (11). Slow dividing cells also have a lower mutation potential associated with the number of divisions (11, 12). Lower mutational potential is associated with resistance to oncogenesis, as well as with a lower number of neoantigens and consequently lower autoimmunity (13). These reasons are important, but in the context of long-living strategies, could there be benefits from keeping stem cells quiescent in the short-term? The quiescence of stem cells, coupled with their metabolic processes, enables them to survive in severely damaged tissues, thereby facilitating regeneration (14, 15). Given their role in regeneration, an increase in the number of stem cells would be expected to enhance the regenerative potential. Stem cells from stroma of adult tissues are maintained at the proportion 10^-5^-10^-4^ (16–21). There should be reasons to maintain this stem cell number at a relatively low level. An interesting note that an increased number of stem cells suggests a lower number of divisions for each, this way significantly reducing a chance of a random cooperation of oncogenic mutations in a single cell, thereby lowering cancer risk (11). A potential explanation is that it is a matter of energy consumption efficiency. However, a tenfold change in the number of stem cells results in a mere 0.1% alteration in the total value. More firm reasons could be derived from the immunomodulatory properties of stem cells. Pronounced and diverse immune modulation of mesenchymal stem cells (MSCs) earlier led to their identification as agents of the immune system (22, 23). However, demonstrated later immune rejection of mesenchymal cells in transplantations ceased that idea (24, 25). Recently, the immune privileges (IPs) of stem cells, including MSCs, have been demonstrated (20, 26–28). It was previously suggested that the IPs of stem cells are associated with their quiescent state and relate to regeneration and inflammation regulation (26, 29). A number of molecular mechanisms are demonstrated to contribute to immune modulation exerted by MSCs, qualifying them as immune modulatory stem cells (IMSCs) (30). That way, IMSCs are cells demonstrating active participation in immune regulation and capability of IPs. I propose a generalized model that functionally links the newly demonstrated IPs to other attributes of IMSCs.

## Functional model

**Figure 1:**
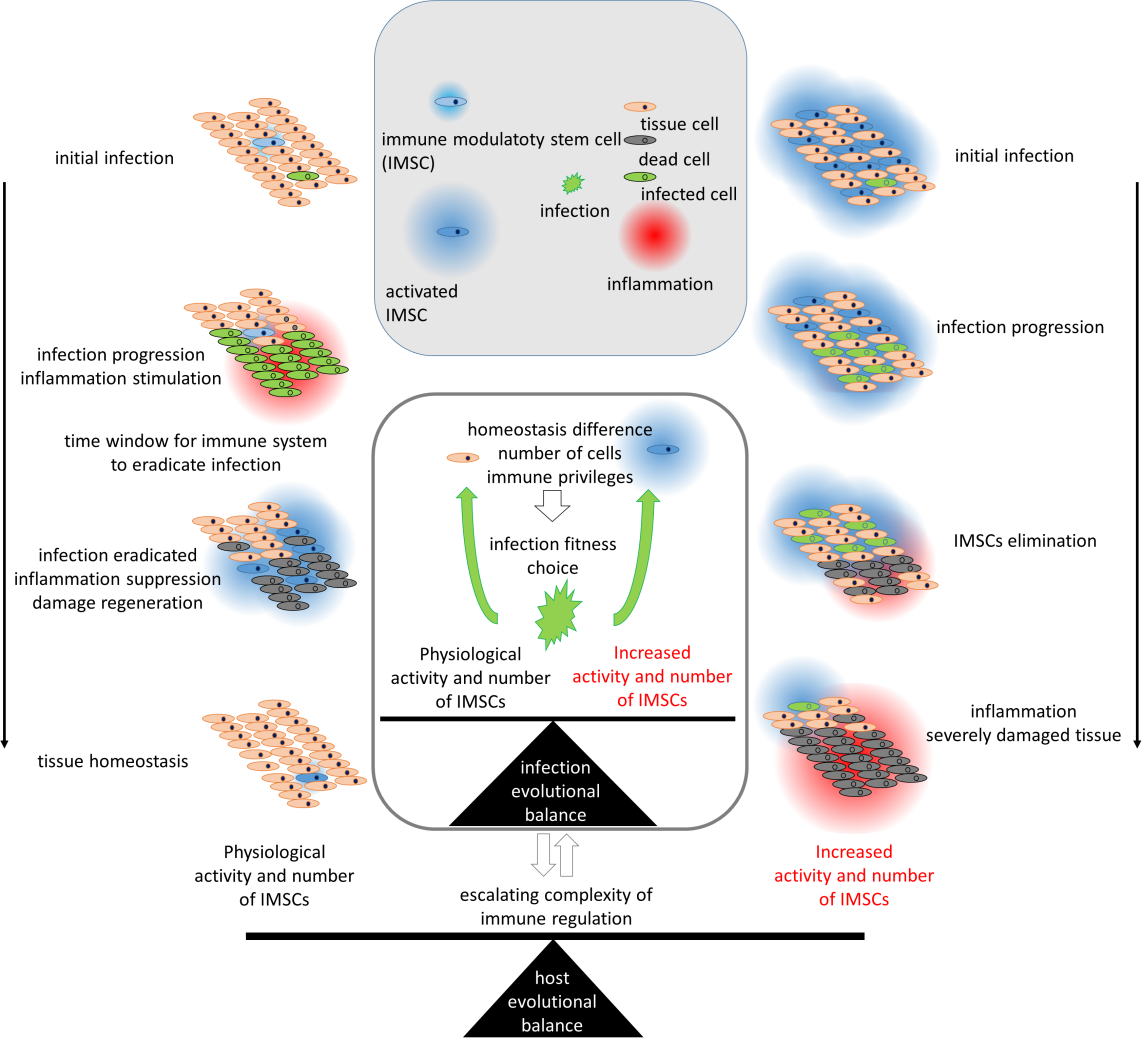
The evolutionary regulation of the activity and number of immune modulatory stem cells (IMSCs) according to their role in the immune system. The left part represents the process of infection under physiological conditions, while the right part represents a hypothetical scenario with increased activity and number of IMSCs. Infection of tissue cells leads to cell death and inflammation of the area. Rare IMSCs, which are able to survive and proliferate in damaged areas due to their homeostatic adaptation, are attracted to the inflamed area. Activated IMSCs suppress inflammation and drive regeneration. Suppressive immune modulation provides protection from the immune system and could be exploited by invading pathogens. The differences in homeostasis of IMSCs protect them from infections. An increase in the number or activity of IMSCs would provide an evolutionary opportunity for infections to adapt to the IMSC phenotype and support the spread of infection, leading to severe tissue damage. Thus, IMSCs and infections are interdependent in the evolutionary equilibrium. The evolutionary competition between host and infection leads to an escalation in the complexity of immune regulation.

The functional significance of IMSCs is of particular evolutionary importance with respect to the stem and immune systems (29). The reports indicate that MSCs not only evade cytotoxic immune action (31), but also actively attract immune cells and can activate or reprogram them depending on the molecular context (32–36). Immune modulation of stem cells is employed in the context of solid organ transplantation and is utilized in the treatment of autoimmune pathology (32–35). This gives ground to mark MSCs as baring functional of immune suppression and as IMSCs. The activation of MSCs and the subsequent induction of the regenerative program results in the suppression of the inflammatory program (34, 37). MSCs have been shown to express a range of immunosuppressing molecules, including PGE2, TGF‐β, HLA-G5, IL‐10, HGF, galectins, CD73, CD39, PD-L1, HLA-G1 and other (30, 34, 37, 38). Immunomodulatory capabilities are more pronounced in IMSCs than in other differentiated cells (39, 40). It is challenging to determine where the immune or other functions of IMSCs are lost during differentiation to their progeny, particularly in light of the potential for dedifferentiation (41, 42). The existing mutual integration of stem and immune systems highlights the evolutionary significance of this integration, as an additional mechanism may potentially act as a break point. This underscores the necessity for evolutionary coordination with respect to the attributes of immune and stem cells involved in this integration. The construction of a comprehensive model is hindered by the vast number of elements and the incomplete knowledge about their connections. Therefore, I propose a functional model (**Figure 1**).

As IMSCs provide immune suppression upon activation (32, 34, 37), they should remain inactive. Otherwise, their immune suppression could potentially compromise the immune protection of a tissue from invading pathogens. A higher concentration of IMSCs results in a more pronounced immune suppression, so reasoning their limited numbers (**Figure 1**). In this manner, IMSCs serve as an activating special agent in the periphery, suppressing the potentially destructive actions of an overactive immune system. This model offers an evolutionary rationale for the maintenance of IMSC quiescence and their low numbers. The traumas and infections have higher risks during life than cancer, so provide possibly stronger selective pressure for long-term living strategies and stay actual for even short-term living strategies.

In the event of infection, resident cells signal to attract immune cells. MSCs are among the cells that signal for immune activation (35, 36, 43). The relatively limited number of IMSCs induces suppressing signals at a slower rate than the initial proinflammatory reaction. This allows the necessary time for an acute inflammatory reaction to occur (**Figure 1**). Upon activation, IMSCs migrate to sites of damage (17, 19, 44, 45), where they exert their immunosuppressive effects. Over time, the inflammatory response stimulates the stem system, thereby inducing its regenerative and anti-inflammatory functions. As a result, the initially inflamed area becomes an area of active regeneration, with the inflammatory response polarized toward a regenerative subtype.

Immune suppression functions can be exploited by invading pathogens (43, 46, 47). The immune system is also responsible for protecting against oncogenesis (48), this emphasizes the risks associated with IPs (46, 49, 50). That way immune suppression should be presented by complex and enigmatic regulation, which serves as a natural barrier against hijacking. Furthermore, the regulatory mechanisms must be robust. The additional protection is provided by a strong connection of this function to a small subpopulation of IMSCs. If pathogens target IMSCs and their immunosuppression, it would be necessary for infection to evolve in order to fit the specific conditions of the stem niche. The physiology and energy exchange of stem cells enable their survival and resistance to infection (51, 52). The fitness of a pathogen to a small subgroup would render it ineffective for the infection of other cells, thereby exerting selection pressure against such fitness (**Figure 1**). The isolation of immune suppression to a small, specific subpopulation of stem cells provides a robust form of protection from infection. The coevolution of immune regulation and infection represents a dynamic and interdependent relationship (46). It is important to note that IMSCs lack absolute protection and may be infected (43, 47). When infected, stem cells can suppress an infection by direct antiviral action and by reducing the number of stem cells through apoptosis (53, 54).

This model also provides a rationale for the seeding of IMSCs to developing tissues during embryogenesis (5). The functional rationale for differentiating between stem cells in adult and embryonic contexts may be attributed to the heightened risk of pathogens invasions in adult tissues during the lifespan. Given the pivotal role of IMSCs in immune function, the divergence in immune status preceding and following labor may provide a potential explanation for the evolutionary adaptation.

The metabolic differences that distinguish stem cells enable them to survive in conditions that would otherwise be lethal for the majority of other cells (15, 51). This enables the regeneration of severely damaged tissues. The model, where IMSCs possess IPs, implies an additional potential for the restoration of areas afflicted by excessive inflammation. As different physiology and a paucity of IMSCs provide evolutionary protection from infection, the risk associated with migration of IMSCs to contaminated tissue is diminished.

## Discussion

The model proposes an evolutionary perspective for IMSCs, including MSC, which have been identified in various tissues of the human body (55). MUSE and VSELs are also stem cells with pronounced immune modulation, derived from a mesenchymal subpopulation of different organs (28, 56, 57). The similarities of functions and molecular mechanisms with other quiescent and immune-privileged stem cells, such as hair follicle stem cells, muscle stem cells or hematopoietic stem cells, require further definition (26, 27). The proposed model does not align with the organizational structure of all tissues and their stem cells. There are examples of stem cell organizations that do not align with the proposed model and that may require significant adjustments (58). The esophageal epithelium is an illustrative example of a tissue wherein 65% of cells are engaged in proliferation, self-maintaince, and repair-related processes, thereby fulfilling the stem cells functions (59). Lgr5^+^ stem cells of the colon and small intestine demonstrate sustained proliferative activity throughout the lifespan (60–62). These cycling stem cells illustrate different evolutionary solutions for tissue-specific mutational processes, in addition to quiescence, to protect against mutations (63). Proliferating Lgr5^+^ stem cells do not exhibit the same IPs as a subpopulation of quiescent Lgr5^+^ stem cells (26). In this manner, the cells also exhibit disparate patterns of immune regulation. The regeneration of acute liver damage is mediated by hepatocytes and biliary epithelial cells. In the context of liver homeostasis, hepatocytes and biliary epithelial cells are in a state of quiescence, yet they undergo activation in response to an acute damage event (64). They are differentiated parenchymal cells of the liver and are the primary contributors to cellular restoration (65, 66). Wound regeneration or inflammation not only activates quiescent cells, but also upregulates dedifferentiation (67). Dedifferentiation may serve to regulate the stem cell pool (68). The number of stem cells is also subject to negative feedback, whereby stem cells inhibit dedifferentiation and reduce the number of surrounding stem cells (69, 70). Further studies are required to elucidate the role of dedifferentiation in immune and stem cell regulation. Further experimental study is required to elucidate the strong functional distinction of quiescent, immune-privileged stem cells. More experiments are required to elucidate the nuances of immune modulation function across stem cells derived from different tissues.

The model provides a logical explanation for the difficulties in expanding stem cells and their immunomodulatory properties used in the clinic to protect against pathological inflammation and immunotoxicity (28, 32, 34, 71–75). The model could be extended to elucidate the IPs of cancer stem cells as an attribute of the stem state (49, 50). The model can also elucidate the role of non-cancerous stroma in the protection of cancer cells by conceptualizing cancerous tissue as a region of active regeneration, wherein the immunomodulatory function of the stem system is activated (67, 76, 77). This provides a natural explanation for the stimulation of immune modulation from non-cancer stroma in response to therapy that damages cancer tissue, thereby further stimulating the function of regeneration (78, 79). The immunomodulatory properties of MSCs are significant and well recognized in the scientific community (32, 34, 71, 73). The principal objective of this article is to designate MSCs or IMSCs as a component of the immune system. It is proposed that IMSCs should be acknowledged as part of the immune system, with a role in the peripheral control of inflammation and autoimmunity, in addition to IMSCs regenerative potential.

The proposed model establishes a functional link between the attributes of IMSCs and their associated IPs and immune modulation. The model provides a functional analysis, eschewing a detailed examination of the underlying mechanisms. A particular mechanism may contribute to different functions simultaneously, thereby forming a complex network. However, it should also exhibit functional robustness beyond this. Additional restrictions imposed on IMSC attributes enhance the overall robustness and offer a compelling explanation for their observed values. To provide a rationale for the links in the model, I present an evolutionary perspective, but with the support of data from experiments that are not necessarily context-specific to evolutionary theory. Nevertheless, the existing deep mutual integration of immune and stem functions provides a robust foundation for the model. It is important to note that the evolutionary link between functions is not necessarily realized by an actual molecular mechanism. Alternatively, functions could be adjusted by independent shifts, which would provide advantages in subsequent generations. The model proposes evolutionary links for the IMSCs attributes. This presentation does not provide a detailed account of the evolutionary process that led to this state or an analysis of the specific mechanisms involved. Nevertheless, these issues warrant further investigation.
